# “The song remains the same”: not really! Vocal flexibility in the song of the indris

**DOI:** 10.1007/s10071-023-01826-6

**Published:** 2023-10-04

**Authors:** Anna Zanoli, Teresa Raimondi, Chiara De Gregorio, Daria Valente, Filippo Carugati, Valeria Torti, Olivier Friard, Longondraza Miaretsoa, Cristina Giacoma, Marco Gamba

**Affiliations:** 1 Department of Life Sciences and System Biology, University of Turin, Via Accademia Albertina 13, Turin, Italy; 2 Parco Natura Viva Garda Zoological Park, Bussolengo, Verona Italy; 3 Groupe d’Étude et de Recherche Sur les Primates de Madagascar (GERP), Antananarivo, Madagascar

**Keywords:** Vocal dimorphism, Distance metrics, Sexual selection, Singing primates, *Indri indri*

## Abstract

In studying communicative signals, we can think of flexibility as a necessary correlate of creativity. Flexibility enables animals to find practical solutions and appropriate behaviors in mutable situations. In this study, we aimed to quantify the degree of flexibility in the songs of indris (*Indri indri*), the only singing lemur, using three different metrics: Jaro Distance, normalized diversity, and entropy. We hypothesized that the degree and the co-variation of the flexibility of indris singing together would vary according to their status and sex. We found that dominant females were more flexible than dominant males when concatenating elements into strings (element concatenation). The number of different elements in a song contribution normalized by the contribution length (contribution diversity) of dominant individuals positively co-varied for seven duetting pairs. Non-dominant individuals were more variable in element concatenation than dominant individuals, and they were more diverse in phrase type than dominant females. Independently from sex and status, individual contributions did not differ in entropy (a measure of the predictability of contributions). These results corroborate previous findings regarding the dimorphism by sex and by status of individual contributions to songs. Thus, they shed light on the presence and expression of flexibility in the behavior of a non-human primate species. Indeed, they potentially show an effect of social features in shaping vocal flexibility, which underlies many communication systems, including human language. We speculate that this degree of flexibility may account for creativity.

**Supplementary Information:**

The online version contains supplementary material available at 10.1007/s10071-023-01826-6.

## Introduction

The concept of creativity in humans often conditions investigations into innovation and inventiveness. If we think of flexibility as a necessary feature of creativity, it is helpful to recall a widespread definition proposed by Sternberg and Lubart (1999). These authors define *creativity* in sports as producing something new, i.e., original, appropriate, useful for achieving a goal (Sternberg and Lubart 1999). This consideration blends well with the framing of studies on animal creativity (Wiggins et al. 2015). Animal creativity does not correspond to human artistic expression but demonstrates adaptive versatility in the communicative repertoire. This fact raises a challenge of investigation: what flexibility traits are shared between humans’ and other animals’ communication?

The pioneering approach of some authors has made it possible to define the boundaries of what we call creativity in an interspecific framework. Flexibility could be seen as a critical prerequisite for creativity, as it can affect the appropriateness of the signal concerning the context or presumed purpose of a given behavioral display (Bailey et al. 2007; Kaufman et al. 2011; Hill et al. 2022). It is also of interest that several creativity studies have focused on play behaviors and social interaction contexts, areas in which the investigation of human creativity has also dwelt extensively (Mitchell 2002; Paulus and Nijstad 2003; Sawyer 2008). Flexibility is a critical feature that enables animals to find practical solutions to situations they have never encountered.

Flexibility in vocal communication can be defined as the capacity to produce new vocalizations or utilize pre-existing vocal patterns in novel ways to accomplish communicative objectives (Arnold and Zuberbühler 2006). This idea acknowledges that animal flexibility may not correspond to human artistic expression, but that flexibility demonstrates adaptive versatility in communicative repertoire (Wiggins et al. 2015). If we narrow our focus to communicative signals, numerous animal species exhibit flexible behaviors that suggest a certain degree of plasticity in vocal communication (Bouchet et al. 2016). For instance, certain bird species incorporate novel elements into their songs, adding new notes or rearranging existing sequences (white‐crowned sparrow: Nelson et al. 2004; zebra finches: Lipkind et al. 2017; Ning et al. 2023; for a review, see Williams 2004). Marine mammals such as dolphins and whales also exhibit individual variation in their vocalizations, suggesting the presence of flexible vocal repertoires in the foraging context (killer whales: Hill et al. 2022) and in captive test sessions (Atlantic bottlenose dolphins: Eskelinen et al. 2016; Kuczaj and Eskelinen 2014).

Moreover, studies on non-human primates have outlined how gestures can produce visual signals that are more flexible and more adaptable to new functions than vocalizations (Call 2008; Pollick and De Waal 2007). Orangutans (*Pongo pygmaeus*) have shown a high level of flexibility, for example, producing novel sounds when allowed to interact with musical instruments (Lameira and Shumaker 2019) or altering the acoustic structure of their calls by putting a hand in front of their mouth (de Boer et al. 2015). Chimpanzees (*Pan troglodytes*) can produce new vocalizations to draw human attention (i.e., used in novel environmental circumstances; Hopkins et al. 2007), and the same has been suggested for gibbons (*Nomascus siki*, Caspar et al. 2020).

However, a different matter might concern the emission of sequences, which are used to encode different information addressed to conspecifics. For example, Arnold and Zuberbühler (2012) showed how putty-nosed monkeys (*Cercopithecus nictitans*) produce two different acoustic types that can chain differently in sequences to evoke behaviors usually observed in the presence of different predators. Furthermore, the ability to concatenate a limited number of elements in a series has been interpreted as an opportunity to encode more information through vocal signals (Engesser and Townsend 2019).

Concatenating elements in different ways also affects species using particular vocalization sequences, such as songs. A song is a series of notes of different types, uttered following a hierarchical structure and characterized by a frequency variation (De Gregorio et al. 2022a). In white-handed gibbons (*Hylobates lar*), for example, songs performed in a duet or in an anti-predatory context show several different characteristics: the percentage of ‘hoo notes’ and ‘leaning wa’ was higher if the song was produced in the presence of a predator and these songs also always contained ‘sharp wow’ notes (Clarke et al. 2006).

Here, we aimed to study phrase and element concatenation in indris (*Indri indri*), the only singing lemur (De Gregorio et al. 2022a), to understand the degree of within-individual and between-individual flexibility. To advertise the occupancy of a territory (Torti et al. 2013, 2018; Spezie et al. 2022), these lemurs produce species-specific songs (Valente et al. 2019, 2022) that can be dominant male–female duets or choruses when also the offspring sing with their parents (Giacoma et al. 2010; Torti et al. 2017). These songs can be heard by humans from more than 2 km away from the emitting group (Gamba et al. 2011; Zanoli et al. 2020), and are recognizable from other types of songs given by the species in different contexts (i.e., territorial encounters or members of a family group disperse during feeding, Torti et al. 2013). Furthermore, songs change during development, and females and males remarkably modify their song structure during ontogeny (De Gregorio et al. 2021a). Adult songs also differ between the sexes, showing that females usually emit more units but shorter in duration, while males give fewer units with longer duration (Giacoma et al. 2010; De Gregorio et al. 2019a; Valente et al. 2021). Songs are rhythmic displays characterized by a specific rhythmic pattern (Gamba et al. 2016; De Gregorio et al. 2019a; Gregorio et al. 2021b).

The songs of the indris are ideal models for studying animal flexibility from both a practical and theoretical perspective. Indeed, duetting behavior has been defined as an observable and significant cognitive investment that signals attention toward a partner. (Kaplan 2023). In this frame, the songs represent an excellent example of contextually homogeneous collective behavioral displays, in which the contribution of each individual can be identified and structurally described. Through this, recent work found that indris can adapt the structure of their songs based on the identity and numbers of co-singers in a chorus (De Gregorio et al. 2022b), but with differences between males and females. Interestingly, while many studies on mammals, including the indri, have found an influence of the animal rank on spectral features of vocal communication (e.g., indris—*Indri indri*, Gamba et al. 2016; baboons—*Papio cynocephalus,* Fischer et al. 2004; mice—*Mus musculus*, Sangiamo et al. 2020; fallow deer—*Dama dama*, Vannoni and McElligott 2008; humans—*Homo sapiens*, Borkowska and Pawlowski 2011), only a few have investigated the link between social status and other vocalization characteristics, such as vocal sequence and usage (Geladas—*Theropithecus gelada*, Gustison et al. 2019; Japanese macaque—*Macaca fuscata*, Lemasson et al. 2013).

Information can be conveyed by the mode of concatenation, predictability, and diversity, so it makes sense to investigate the variation in each of these variables in the composition for the individual contribution. We used three measures: (1) logical distance (e.g., how different the sequences are; Zanoli et al. 2020), (2) diversity (how many types of elements we have in the sequence) normalized to duration, and (3) a measure of entropy (a measure of the randomness of a sequence; Kershenbaum 2014). Thus, we considered flexibility in terms of variation, diversity, and entropy of the song structure. We hypothesized that individuals participating in singing might show a different degree and covariation of flexibility during singing according to sex and social status. Studying how these vocal displays can covary help unravel the complex interactions and dependencies among individuals within a social group. This analysis provides a framework for comprehending how the flexibility of individual contributions to songs is a feature that contributes to the emergence of group-level patterns (Briefer et al. 2011). Indeed, examining the covariation structure makes it possible to identify shared behavioral tendencies and key influencers within the group and shed light on the mechanisms driving social dynamics (Strandburg-Peshkin et al. 2018; Ioannou and Laskowski 2023). For instance, the sequence similarity analysis within and between groups and individuals demonstrated that meerkats sequences showed higher within-individual consistency in adults vs. subadults and females vs. males (Rauber et al. 2020). In the same study, Rauber and colleagues (2020) showed that a graded sequence system may convey contextual information to conspecifics, while other features may encode information about caller identity.

We formulate three predictions from these pieces of evidence and previous knowledge about the indris. We predict that dominant females will show greater flexibility than dominant males (Prediction 1), that there will be a positive covariation in flexibility between duetting partners (Prediction 2), and that dominant indris (the reproductive pair) will show greater flexibility than non-dominant individuals (Prediction 3).

## Materials and methods

### Data collection and acoustic analyses

We recorded spontaneous songs of indris living in the Maromizaha New Protected Area (Madagascar, 18°56′49′′ S, 48°27′53′′ E). Songs were recorded during a long-term data collection from 2010 to 2020, using solid-state recorders (Olympus S100 and LS05, Tascam DR-100, DR-40, and DR-05, Zoom H5) equipped with shotgun microphones (Sennheiser ME 66 and ME 67) at a sampling rate of 44.1 kHz and 16-bit resolution. We assigned each contribution to its emitter via the focal animal sampling method (Altmann 1974) and identified singers through natural marks while looking at them during singing. Songs could be duets of reproductive pairs, or duets as part of choruses in which dominant individuals sang with their offspring. We recorded 599 songs composed of the contributions of nine reproductive females, nine reproductive males (hereafter defined dominants) and 21 non-reproductive males and females (hereafter, non-dominants) from eight familiar groups. We considered the individuals in the groups as non-dominants, except the reproductive pair, independent of their age (1–6 years). Therefore, the number of groups and dominant reproductive individuals are different (eight vs. nine), since a dominant reproductive male and a dominant reproductive female of two groups (3MZa and 5MZa) changed during the data collection period. In the analyses, we considered the groups 3MZa and 5MZa as different groups, thus resulting in 10 groups analyzed.

Following Zanoli et al. (2020), we performed the acoustic analysis editing indri songs using Praat 6.1.40 (Boersma 2001). First, we created a spectrogram for each record and saved the singer’s identity information into a Praat TextGrid. We then extracted each individual contribution and labeled the vocal units as long notes (LN), single units (SU; De Gregorio et al. 2019a, b), and descending phrases (DPs; sequences of adjacent notes with a descending frequency pattern composed of 2–6 units. Valente et al. 2021; Torti et al. 2013). Given that the bulk of the indris’ songs is based on descending phrases and single units, for the statistical analysis, we considered only single units (SUs) and phrases composed of two (DP2), three (DP3), four (DP4), five (DP5), and six (DP6) units (Sorrentino et al. 2012; Gamba et al. 2016; Torti et al. 2017).

### Distance calculation

We used the Jaro string Distance (hereafter JD; Jaro 1989) to assess variation in the indri song structure according to sex and status (dominant vs. non-dominant). We isolated individual contributions from each song composed by the concatenation of SUs and DPs. Then, we transformed each contribution into a string of phrase types (e.g., SU|DP3|DP2|DP3), thus obtaining a representation of the concatenation of the phrases uttered in each contribution (Zanoli et al. 2020; Harrington 2016). From the 599 songs, we extracted 566 strings for dominant males, 599 for dominant females, and 363 for non-dominants. We did not consider non-dominants’ sex to maintain a numerically comparable dataset with dominants. We computed a distance matrix calculating the JD in R (*stringdist*; van der Loo 2014) from the strings extracted from each contribution. This allowed us to obtain a square matrix (1528 × 1528) in which we then identified rows and columns with labels (*N* = 39) comprising the identity, sex (only for dominant individuals), and status of each individual (dominant male, dominant female, non-dominant). The JD is a simple but elegant string metric initially developed to detect text similarity (Fellegi and Sunter 1969) and later applied to detect differences in behavioral sequences with robust results (Oakley 2020). The JD algorithm calculates the number of transpositions and matches between two strings and assigns a distance between 0 (no matches) and 1 (complete match). High pairwise JDs indicate high similarity between the strings, while low JDs denote low similarity (Jaro 1989). In our case, greater flexibility would be indicated by lower JD scores. Therefore, to understand which category (Dominant males, Dominant females, Non-dominants) was more flexible in concatenating of elements into sequences we transposed the matrix into a data set in which each row represented a JD distance, the dyad of individuals between which the JD was calculated, and their sex, status, and group. We categorized each distance (hereafter, distance type) as "within" when calculated between two contributions from the same individual or "between’’ when calculated between contributions from different individuals. Finally, we ran a Linear Mixed Model (LMM, *lme4*; Bates et al. 2014) by setting the square root of the JDs as the response variable, a factor combining the distance-related features (distance type—sexes of the dyad—status of the dyad) as a fixed effect, and the dyad identity as a random factor. We used the square root transformation to obtain a Gaussian distribution of both response variables (*fitdistrplus*; Delignette-Muller and Dutang 2015) and model residuals (*diagnostic.plot*; Estienne et al. 2017). We used a likelihood ratio test (*Anova*—“Chisq” test argument; Dobson 2002) to test the significance of the model, comparing it against a null model comprising only the random factor (Forstmeier and Schielzeth 2011) and to estimate the *p* values of each predictor (*drop1*; Barr et al. 2013). We summarise the data processing steps and distance calculation procedure in Fig. SM1.

We employed a Support Vector Machine (SVM) with the kernel method as a classification algorithm to investigate whether it was possible to identify differences between the sexes from JD values. Finding a significant classification between the sexes via SVM would mean that flexibility in the concatenation of elements in sequences is sexually dimorphic. We used the Support Vector Machine only on strings generated from dominant contributions, because the LMM model had already shown a difference between dominants and non-dominants. Moreover, changes during development may potentially influence the emissions of non-dominants (De Gregorio et al. 2021b). Although extensions have been developed for multi-class classification, SVM was initially intended for two-class classification problems. We used a competitive strategy to determine the best model by tuning parameters using the correct classification rate. Then, parameters C and Sigma of the best-ranked model using tenfold cross-validation were used to rerun the SVM on the full dataset and evaluate the overall accuracy. We then annotated the correct classification rate of the training set (consisting of 70% of the data set) and the testing set (the remaining 30%).

### Normalized Diversity

To investigate differences in the flexibility of indri songs among sexes or status, we calculated an additional variable for each individual contribution, the normalized diversity. We normalized diversity by dividing the number of different phrases and standalone elements composing an individual’s contribution by length (the number of phrases (DP) and standalone elements (SU) composing an individual’s contribution). For example, for DP2|DP3|DP3|DP5|DP4, we would determine a diversity of 4 (DP2, DP3, DP5 and DP4), which was then divided by the length (5, as the number of elements composing the string), resulting in 0.8, corresponding to the normalized diversity. We then ran a LMM (*lme4*; Bates et al. 2014), testing whether the normalized diversity of vocal contributions differed among dominant males, dominant females and non-dominants. Normalized diversity, considering the number of types of elements included in a contribution, can indicate how distinctive an individual is in a particular song. In the model, we used singer identity and song ID as random factors to control for the singers’ identities and the particular song they were participating in. We used the logarithmic form of normalized diversity to reach the normal distribution of both the response variable (*fitdistrplus*; Delignette-Muller and Dutang 2015) and model residuals (*diagnostic.plot*; Estienne et al. 2017). We tested the significance of the model and estimated the *p* values for predictors as described in the previous paragraph.

Then, to test for a possible covariation of the normalized diversity between dominant co-singers belonging to the same group, we used Spearman’s correlation test (R function *cor.test*, method = ‘spearman’; R Core Team 2022) comparing, within each group, the normalized diversity of contributions of dominant males and dominant females when duetting. High levels of covariation would mean comparable levels of flexibility between sexes, while low levels of covariation would mean sexually dimorphic flexibility patterns. We used the Spearman’s correlation test (non-parametric) as the normalized diversity of each individual was not normally distributed (tested with the Shapiro–Wilk normality test, R function *shapiro.test*; R Core Team 2022).

### Entropy rate

Vocal sequences can be structured as a sequence of vocalizations emitted by an individual over a given time span. A sequence of vocalizations can be modeled as a stationary time-homogeneous Markov chain (i.e., an indexed sequence of random variables; Cover and Thomas 1991, 2006), where the unpredictability of an individual’s vocal sequence can be quantified by the entropy rate of the process (Kershenbaum 2014; Vegetabile et al. 2019). Hence, the entropy rate of a discrete-state stochastic process quantifies the predictability of the next observation given both the history of observations which occurred before it, and the stationary distribution of the process (Vegetabile et al. 2019). The latter can be defined as the asymptotic proportion of time that the Markov chain will spend in any state of the process (Levin and Peres 2017). Regarding vocal flexibility, the higher the entropy rate (higher unpredictability), the higher the flexibility.

We modeled each indri song contribution as a discrete-state stochastic process (in our case a Markov chain composed of a sequence of descending phrases and single units; Cover and Thomas 1991, 2006), and we quantified the predictability of individual contributions’ composition through the Markov entropy rate of the process (Kershenbaum 2014). We computed the conditional probabilities of transition between descending phrases (DPs) and single units (SUs) emitted in each individual’s contribution (i.e., conditional entropy; Levin and Peres 2017) and summarized them into an empirical transition matrix using Behatrix software (version 0.9.13; Friard and Gamba 2021). Each transition matrix defines the dependence structure of a contribution. We then developed first-order Markov chains on a vector of random variables (Vegetabile et al. 2019) with (a) variables corresponding to the number of elements (DPs and SUs) observed in the individual contributions and (b) the probability of transition between variables, corresponding to the empirical transition matrix probability (Vegetabile et al. 2019). By employing the R package *ccber* (Davis et al. 2017), we simulated a Markov process to determine the entropy rate value for each individual. In this process, the entropy rate is calculated as the weighted average of the conditional entropy of an element in the sequence given the previous element, where the weights are given by the stationary distribution (Levin and Peres 2017).

We conducted a Kruskal–Wallis rank sum test to investigate potential differences in entropy rates between sexes and social status (using the *kruskal.test* function in R; R Core Team 2022). Finally, we used Spearman’s correlation (*cor.test*, method = ‘spearman’; R Core Team 2022) to examine the correlation of individual entropy rates between the dominant male and dominant female indris. Finding a positive correlation between the sexes would imply that flexibility in one individual’s contribution may be associated with flexibility in the output of an individual of the opposite sex, thus showing a tendency to exhibit a similar level of unpredictability. Conversely, a negative correlation would imply a tendency toward sexual dimorphism.

## Results

### Song structure variation

**Fig. 1:**
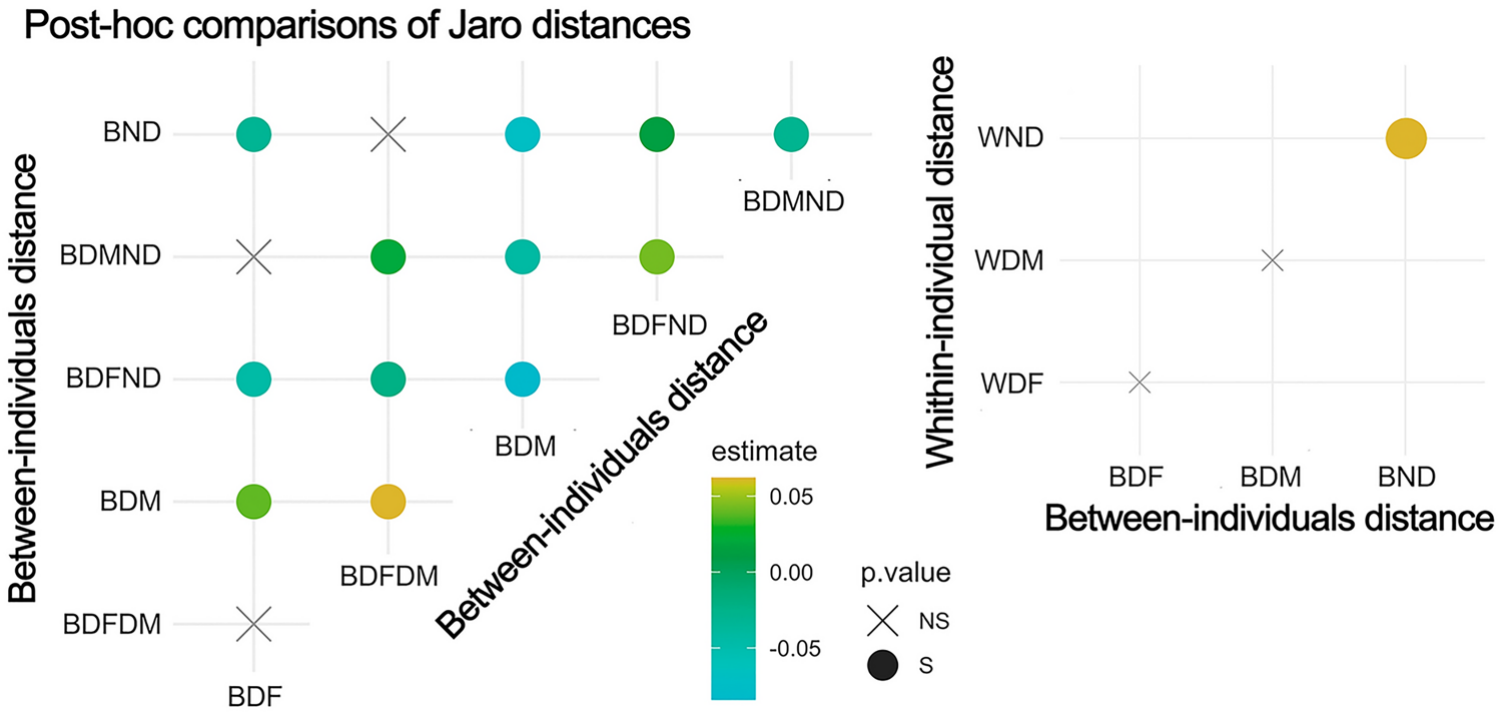
Matrix plots displaying the results of the post-hoc comparisons in Table SM1 testing the effect of distance type (within or between-individuals distance per sex and status) on the Jaro Distance. On the left, the post-hoc comparisons of between-individuals Jaro distances per sex and status categories (B = between, D = dominant, ND = non-dominant, F = female, M = male). On the right, the matrix plot shows the post-hoc comparisons of within-individual vs. between-individual Jaro distances per sex and status categories (B = between, W = within, D = dominant, ND = non-dominant, F = female, M = male). The comparisons in both plots should be read as an *x*-axis (rotate of 45° in the left panel) label vs. a *y*-axis (vertical one) label. The *X* symbol displays non-significant (*p* > 0.05) comparisons, and the colored dot shows significant comparisons (*p* < 0.05), with the number indicating the value of the estimate

We found that the variability in concatenating single elements and phrases, expressed as Jaro Distance, varied according to the sex and the social status (LMM—full vs. null model: χ^2^ = 253.462, df = 8, *p* value < 0.001). The results of the Tukey–Kramer test are shown in Fig. 1 and Table SM1.

When looking at the inter-individual level, pairwise comparisons (Table SM1) highlight that Jaro Distance between dominant females (BDF) was higher than JD between dominant males (BDM) but lower than JD between non-dominants (BND). In addition, JD between dominant males (BDM) was lower than between non-dominants (BND). These results show that, regarding flexibility in the concatenation of elements in sequences, non-dominant individuals possess the highest degree, followed by dominant females and last by dominant males.

When looking at the within-individual level, pairwise comparisons (Table SM1) highlight that, for both dominant males (WDM) and dominant females (WDF), the variability between individuals did not differ from the within-individual one. On the contrary, the variability between non-dominants was higher than their within-individual one (WND). Non-dominant individuals show the highest degree of vocal flexibility at the within-individual level, thus considering flexibility across their contribution to the songs.

When classifying the sequences of dominant females, dominant males, and non-dominant indris obtained using the JD distance matrix, we got an above-chance average classification rate of 64.4% (Training error: 0.016367). In particular, the algorithm correctly classified 76% of dominant female sequences, and the remaining cases were assigned to dominant males and non-dominants for 18% and 6%, respectively. Half of the dominant male and half of the non-dominant sequences were correctly classified (53% and 48%, respectively), with the rest being equally misclassified between the other two categories. The high rates of correct classification imply that the flexibility in the concatenation of elements in sequences is sexually dimorphic and allows potentially recognizing dominants from non-dominant indris.

### Normalized Diversity and contribution flexibility

**Fig. 2:**
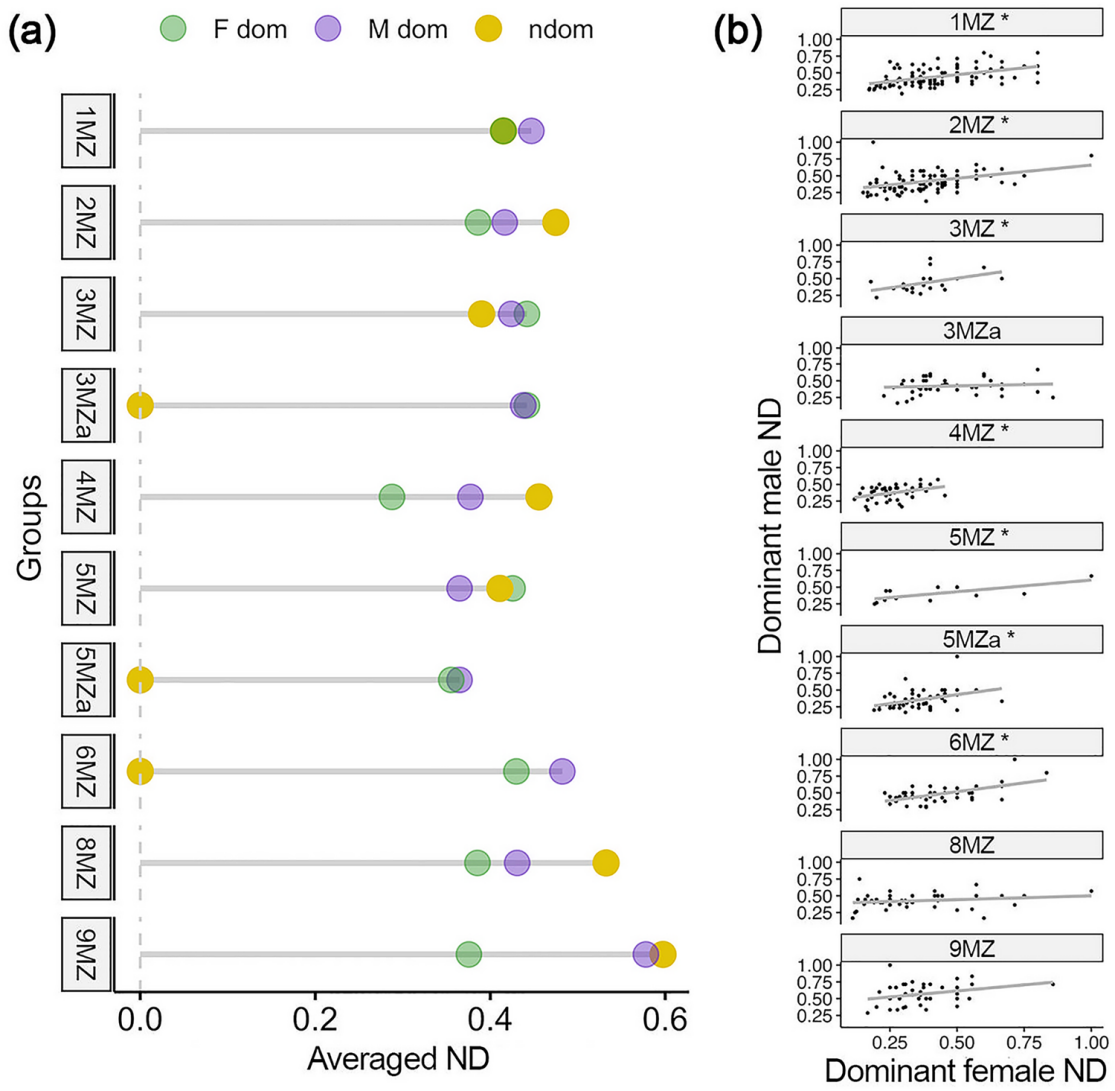
**a** Lollipop plot showing the Normalized Diversity (ND, averaged per sex, status and group) for all the indri groups in the sample. **b** Regressions between the Normalized Diversity (ND) values of cosinging dominant male (*y*-axis) and dominant female (*x*-axis). The asterisk indicates the groups showing a significant positive correlation between ND values

When testing whether the flexibility of the cosingers co-varies during the duetting, we found an overall effect of sex and status (*F*_2,30.469_ = 5.502, *p* value = 0.009; Table SM2, Fig. 2a), but, when looking at post-hoc comparisons, normalized diversity did not significantly differ between dominant females and dominant males (Tukey–Kramer test, estimate = −0.156, SE = 0.088, Kenward–Roger df = 33.2, t.ratio = −1.767, *p*_adj_ = 0.196), and dominant males and non-dominant indris (Tukey–Kramer test, estimate = −0.096, SE = 0.079, Kenward–Roger df = 38.0, t.ratio = −1.211, *p*_adj_ = 0.454). Instead, dominant females significantly differed from non-dominant indris (Tukey–Kramer test, estimate = −0.252, SE = 0.079, Kenward–Roger df = 38.1, t.ratio = −3.179, *p*_adj_ = 0.008), showing the highest flexibility degree in non-dominant individuals (Normalized Diversity_dominant females_ = 0.377土 0.141; Normalized Diversity_dominant males_ = 0.376土 0.146; Normalized Diversity_non-dominants_ = 0.381土 0.149).

Testing each group individually, we found a significant correlation of the normalized diversity between each dominant male and dominant female in seven pairs, but not in three pairs (3MZa, 8MZ, 9MZ). In particular, the seven pairs showing a significant test displayed a positive correlation (Fig. 2b). The results of Spearman’s correlation tests for each group are shown in Table SM3. The covariation of the normalized diversity thus shows an overall mixed pattern among the duetting indris.

### Contribution complexity and predictability

**Fig. 3:**
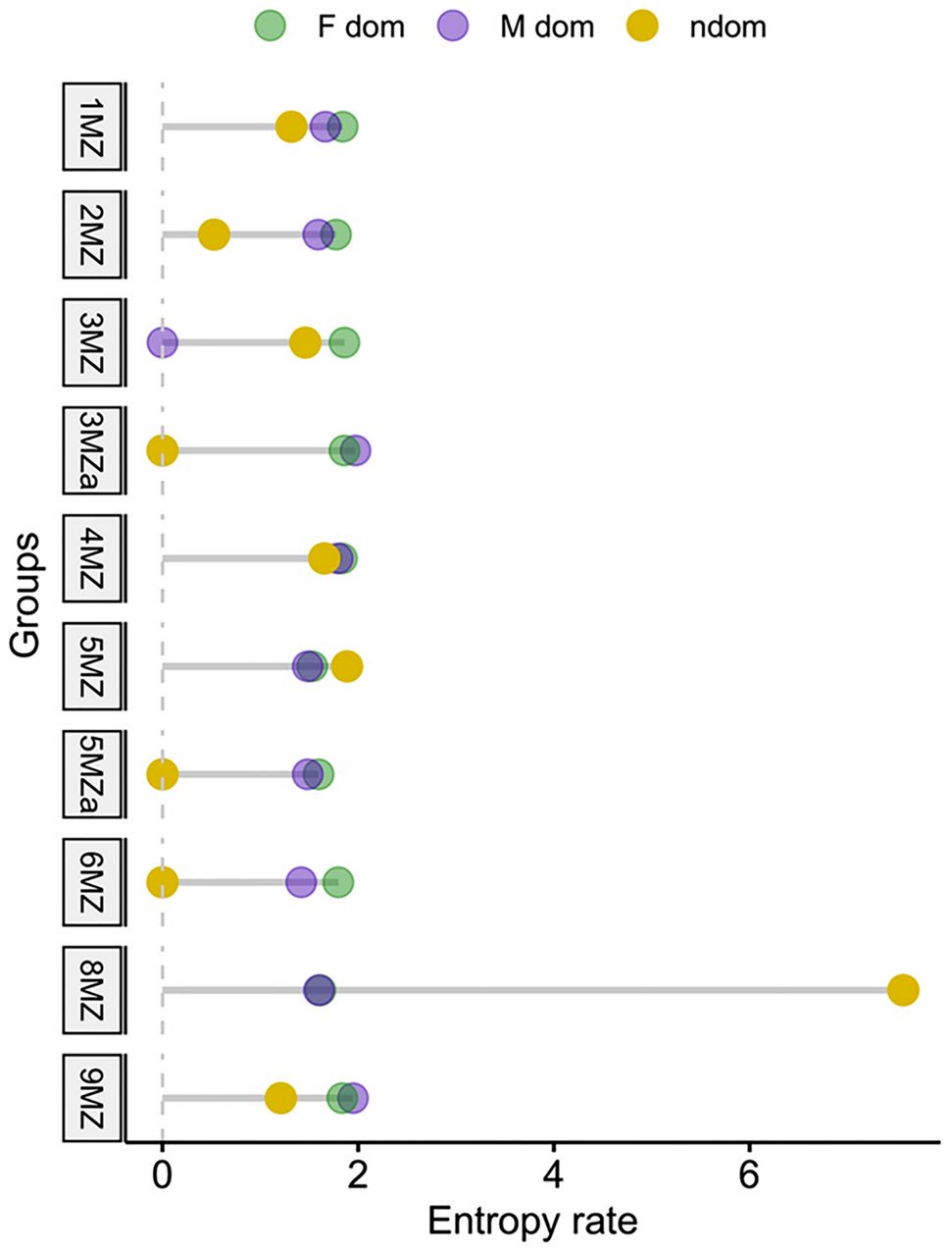
Lollipop plot showing the entropy rate (averaged per sex, status and group) for all the indri groups in the sample

We did not find a significant difference in entropy rates among sexes and status (Dominant Male, Dominant Female, Non-dominant; Kruskal–Wallis, χ^2^ = 4.774, df = 2, *p* value = 0.092; Table SM4, Fig. 3). Similarly, we found no significant correlation between the entropy rates of dominant males and the entropy rates of dominant females (Spearman’s correlation test, Rho = 0.335, *N* = 10, *p* value = 0.335). This result shows that higher flexibility in the singing of a female does not imply higher flexibility in male contribution, and vice versa.

## Discussion

We studied element and phrase concatenation in the indris participating in advertisement songs to understand the degree of within-individual and between-individual flexibility. The results are discussed below following the structure of our three predictions: dominant females should show greater flexibility than dominant males; the covariation in flexibility between duetting partners should be positive; dominant indris (the reproductive pair) show greater flexibility than non-dominant individuals.

### Flexibility between dominant males and dominant females

We predicted that dominant females would show greater flexibility than dominant males and found partial support in our results. The kernel Support Vector Machine on the Jaro Distances of dominant individuals allowed us to discriminate sexes by considering the element concatenation of the individual contributions. Indeed, dominant females showed higher correct classification rates than dominant males, confirming their highly distinctive sequences in agreement with previous findings in the indris (Zanoli et al. 2020) and in meerkats (Rauber et al. 2020). Conversely, as shown by the Markov entropy rates, the complexity and the predictability of the indris’ contributions are likely unrelated to the sex of the individual. Specifically, we predicted that dominant female individuals showed greater flexibility than dominant males, and in fact, we found that dominant females showed a higher degree of between-individual variability in concatenating single elements and phrases than dominant males. However, contributions’ normalized diversity did not differ between sexes. These results partially support our first prediction, because, compared to dominant males, on one hand, dominant females are more flexible in element concatenation, but, on the other hand, the two sexes do not differ in the flexibility expressed through the normalized diversity. The lack of statistically significant differences in the normalized diversity can be attributed to several factors intimately linked to the mathematical characteristics of this parameter and the biological process it describes. First, we must consider that the variation of the string on which we calculate diversity has a minimum of 1 and a maximum of 6 (SU, DP2, DP3, DP4, DP5, DP6; Sorrentino et al. 2012; Gamba et al. 2016; Torti et al. 2017) phrase types for both males and females: the number of combination possibilities of a limited number of phrase types obviously represents a constraint in the total diversity. Moreover, the first phrases or notes that are concatenated at the beginning of the song are usually short (e.g., SU, DP2, DP3; Pollock 1986), so they vary over an even smaller number of values, and this could critically influence the value of Normalized Diversity on songs of short duration. To this, we must add that the parameter is expressed as a ratio (the denominator of which is the duration of the contribution considered). Together, these two aspects could determine the lack of quantitatively significant differences between the sexes for normalized diversity.

Our findings align with a previous work investigating the phrase combinatorics only in dominant indris (Zanoli et al. 2020) and the differences in contribution between sexes (Giacoma et al. 2010). The corroboration of Zanoli et al. (2020) results acquires more relevance when considering the distance metrics used. Although the Levenshtein (used in Zanoli et al. 2020) and the Jaro distances consider different parameters when calculating the similarity between two strings (Margoliash et al. 1991; Jaro 1989), both algorithms gave robust results when applied to indri songs and showed that dominant females are more flexible than dominant males when concatenating elements into strings, bearing in mind that non-dominant individuals show the highest flexibility degree. These results corroborate previous work that suggested that dominant females contribute to the indris’ songs (De Gregorio et al. 2019a, b) and that they are more flexible in notes’ spectro-temporal parameters than dominant males (Torti et al. 2017). Although the reproductive couple has a higher rank than that of other group members (usually offspring; Pollock 1975), reproductive females seem to be dominant over reproductive males in terms of access to resources (Pollock 1979; Kappeler and Pozzi 2019), and this could reflect in their songs’ structure. This is similar to what was observed in geladas, where leader males perform more complex call types and have longer sequences than follower males (Gustison et al. 2019). In Japanese macaques, rank explained differences between adult males in contact call usage (Lemasson et al. 2013). In addition, high-ranking males performed longer syllables in yellow baboons, which has been considered a possible indicator of males’ stamina (Fischer et al. 2004). Boosted complexity and length in the individual contributions of these species have been proposed to serve as an honest signal of the emitter’s quality, since calls are subject to sexual selection. In this sense, previous literature shows that the flexible modulation of the structure of the signal and its length is particularly adaptive in those sex, age and status categories that need to advertise their quality to potential partners.

Our results suggest that, in indris, dominant females, being more flexible in element concatenation, are more creative than dominant males if we consider flexibility as a necessary correlate of creativity (Sternberg and Lubart 1999). The role of dominant females during singing is critical to understanding these results. Indeed, dominant females show more creative potential than dominant males not only in the element concatenation but also by adjusting the rhythmic and temporal structures of their contributions according to the number of singers in the chorus (Gamba et al. 2016; De Gregorio et al. 2019a, b; De Gregorio et al. 2022b). As suggested for white-handed gibbon songs (*Hylobates lar*), the temporal coordination of a duet relies on the individuals’ capability to adjust their contribution to that of the mate temporally (Terleph et al. 2018). Terleph and colleagues proposed that, for turn-taking to occur effectively, males would be the ones adjusting their song to their mate’s one because of females’ noticeable variation in spectral and temporal characteristics of their phrases (Terleph et al. 2018). Our results did not corroborate the pattern suggested by Terleph and colleagues (2018) but are in line with previous findings on Northern white-cheeked (*Nomascus leucogenys*, Deputte 1982), agile (*Hylobates agilis,* Koda et al. 2013), white-handed gibbon (*Hylobates lar,* Raimondi et al. 2023) and indri (Torti et al. 2017; De Gregorio et al. 2019a, b). In indris, the dominant pair generally orchestrates the overall architecture of the duet: the duet between the reproductive pair is the most common type of singing organization (De Gregorio et al. 2022a, b), only occasionally succeeded by non-dominants, consisting of the offspring, and shows longer durations and higher degrees of overlap (Gamba et al. 2016). In the present case, in particular, dominant females’ higher flexibility than dominant males seems to drive the song “template” by adjusting their contributions to maintain the song structure despite the less flexible and stereotypical nature of male contributions (less flexibility in element concatenation). This aspect should be viewed as complementary to the fact that non-dominant individuals show an even higher degree of flexibility. The following sections explore the biological reasons behind this finding in more detail.

### Flexibility covariation between duetting dominant males and dominant females

Our second prediction indicated that the flexibility of two duetting dominant individuals should covary (i.e., higher degree of flexibility of an indri should match a higher degree in the duetting partner). Our results partially support Prediction 2, because we found that the diversity of the dominant individuals positively co-varied only for seven out of ten duetting pairs. On one hand, for seven pairs, a high diversity of the female corresponds to a high diversity of the male, and vice versa. On the other hand, for three more pairs, we did not find a statistically significant association. These results remarkably extend previous findings that centered on the sexually dimorphic features of the indris’ duetting. Indeed, female contribution in indris is critical to determine the total song duration (Giacoma et al. 2010), influences the duration of males’ contribution (De Gregorio et al. 2019a, b), and shows higher flexibility in concatenation (Zanoli et al. 2020). Further studies may elucidate whether and how the relationship we found in terms of contribution diversity between males and females could be linked to a (or to a lack of a) non-random phrase type combination, where two duetting partners could use a statistical association between the phrase types they use (Logue and Krupp 2016). Indeed, while the lack of non-random phrase type association may indicate scarce support for the existence of answering rules, a causal relationship (either negative or positive) may entail the existence of governing rules, with several potential explanations (Logue and Krupp 2016). Interestingly, the three pairs for which we found no significant co-variation are those living in larger groups of up to five individuals. The larger the groups, the higher the possibility that youngsters join the parents’ singing, whose structure might, in turn, be disregarded (De Gregorio et al. 2022b). Hence, our findings also align with potential reduction of singing complexity related to the presence of youngsters within the groups (De Gregorio et al. 2022b). We cannot exclude that females or males in turn can decrease the diversity of their contribution to facilitate subadult singing (De Gregorio et al. 2022b).

### Flexibility between dominant and non-dominant individuals

Finally, we predicted that dominant indris (the reproductive pair) would show greater flexibility than non-dominant individuals. We rejected Prediction 3 as non-dominant individuals were more flexible in element concatenation than dominant males and dominant females. In addition, they were more diverse in phrase type than dominant females but did not differ from dominant males. Moreover, the complexity and the predictability of indris’ contributions estimated with the Markov entropy rates seem not to be related to the status of the individual.

Although we cannot exclude a possible effect of age and sex on the results of this investigation, as songs are known to change during ontogeny (De Gregorio et al. 2021a) and to depend on sex (Giacoma et al. 2010), half of the non-dominant indris were adults. In addition, even though dominant male contributions are known to be less plastic than dominant female ones (Zanoli et al. 2020; De Gregorio et al. 2019a, b) and more similar to their offspring contributions (Torti et al. 2017), our results from the Kernel Support Vector Machine highlight that the contributions of dominant males and non-dominants do not overlap in terms of element concatenation. The data showing greatest variability in non-dominant indri agrees with the findings collected on the sequences of meerkats, where the authors observed less variability in females and adult males (Rauber et al. 2020).

Future work might assess if there are differences in song flexibility also between non-dominant males and non-dominant females: previous work found that the differences in non-dominant juvenile male and non-dominant juvenile female indris during ontogeny affected mainly the song’s temporal parameters and to a lesser extent spectral parameters (De Gregorio et al. 2021a), but the differences between non-dominant adults individuals remain unexplored. At the same time, social status may also play a role in determining the flexibility of non-dominant indris. It has been observed, for instance, that social status and the increased competition pressure for bachelors and not resident individuals, affect progression trends in the syntactic structure of signals of rock hyraxes, *Procavia capensis* (Demartsev et al. 2019).

The relationship between the quality of individuals and flexibility in indri remains uncertain. However, a similar mechanism may influence status-related differences in element concatenation and diversity. Non-dominants contribute more diverse vocalizations than dominant females, potentially due to the latter’s role in song organization and dominance over the group (De Gregorio et al. 2019a, b; Gamba et al. 2016; Zanoli et al. 2020). Indeed, reproductive females seem to be dominant over all the other members of the group (Pollock 1979; Kappeler and Pozzi 2019) and to orchestrate the general organization of a song in terms of phonation (De Gregorio et al. 2019a, b), rhythm (De Gregorio et al. 2019a, b; Gamba et al. 2016), and phrase combinatorics (Zanoli et al. 2020). Dominant males and females display greater vocal overlap, constrained by reciprocal coordination (Gamba et al. 2016). In this respect, dominants are constrained to engage in reciprocal coordination by adjusting the overlap in the chorus. Free from such constraints, non-dominants exhibit more flexible phrase combinatorics and greater diversity (De Gregorio et al. 2022b). Creativity in non-dominant indri individuals may be advantageous during their dispersal phase when seeking a sexual partner and forming monogamous pairs in new territories.

Flexibility can decline on different scales, for example, within and between individuals belonging to different sex and status categories: therefore, we tested for between- and within-individual differences, finding a high degree of individuality only in non-dominants. This result aligns with previous speculation that non-dominants are more competitively motivated than dominants (Demartsev et al. 2019). Individuality and flexibility appear to play a role in sexual selection mechanisms (Bradbury and Vehrencamp 2003; Robinson et al. 2019), and in indri are expressed through the temporal and spectral characteristics of the contributions of dominants (Giacoma et al. 2010; Gamba et al. 2016; De Gregorio et al. 2019b), and through element concatenation in non-dominants. It is worth mentioning that, unlike other distance metrics (e.g., Levenshtein Distance, Zanoli et al. 2020), the Jaro Distance algorithm also considers the string length. This can explain the lack of pronounced individuality in dominant individuals, which did not differ in phrase type diversity (Zanoli et al. 2020).

Overall, the results presented in this study and those in the literature can be evidence of vocal flexibility in problem-solving (i.e., a measure of creativity). Indeed, we know from the literature that the structure of the indri songs conveys context-specific information through their overall duration: advertisement songs are longer than cohesion ones; in other words, songs with the most prolonged duration are required to effectively defend the territory (Torti et al. 2013). We also know that the contribution of females is crucial in determining the song time frame, and their contribution changes flexibly depending on the number of individuals participating in the chorus (De Gregorio et al. 2019b). In this context, the distinct flexibility of the dominant females compared to dominant males revealed by our results can be seen as a problem-solving ability: being flexible and adapting the contribution to each new chorus (problem-solving to novel situations; Rowell et al. 2021) determines successful singing and, thus, efficient territorial defense. This is in line with findings from human and non-human species on the creative use of vocalizations to solve problems: for example, in human collaborative problem-solving, utterances to elicit and maintain interactions, thus increasing the social bond, were frequently observed (Johnson and Johnson 1986; Yokozuka et al. 2021). Chimpanzees have the necessary socio-cognitive skills to naturally develop a simple communicative strategy to ensure coordination in a collaborative task (Melis and Tomasello 2019). Bottlenose dolphins are well-known for cooperating extensively in the wild and can adjust vocal signals to facilitate the successful execution of coordinated, cooperative actions (King et al. 2021).

Our study demonstrates how different metrics can describe flexibility in individual indri song sequences, for which we detected remarkable variation within- and between-sex for dominants and between-status. We observed this across acoustically distinct but functionally homogeneous songs used for territorial advertisement. Our findings partially agree with observations on other mammals, where changes in call order encoded predator information or risk of predation (Arnold and Zuberbühler 2006; Rauber et al. 2020). In the case of the indris, our flexibility measures indicate consistent variation within vocal displays that solves the same problem. We speculate that this variation may account for creativity, intended as individual variability within a display that still reaches a precise goal (O’Hearn et al. 2015). Our speculations clamor for new studies that could show how the individual’s behavior somehow influences the combination of phrases and units in singing (indri can eat or monitor the environment before and during singing, for example). The hypothesis that an individual’s singing may influence the choice of a particular element or phrase by the individual with whom he or she is duetting is also suggestive but entirely speculative, but only new and more extensive sampling will be able to shed light on these aspects.

### Supplementary Information

Below is the link to the electronic supplementary material.

Supplementary file1 (XLSX 15 KB)

Supplementary file2 (DOCX 257 KB)

## Data Availability

The data sets generated during and/or analysed during the current study are available in the Zenodo repository, https://doi.org/10.5281/zenodo.7473226
