# Supplementary material for: “The song remains the same”: not really! Vocal flexibility in the song of the indris
Source: Anim Cogn. 2023 Oct 4;26(6):2009–21. doi: 10.1007/s10071-023-01826-6 (PMC10769932; doi:10.1007/s10071-023-01826-6)
Supplement: Supplementary file 2 — Supplementary file2 (DOCX 257 KB) [file 10071_2023_1826_MOESM2_ESM.docx]

**Supplementary material**

article title: **“The song remains the same”: not really! Vocal creativity in contribution flexibility in the indris**

journal name: Animal Cognition

author names: Anna Zanoli^1*°^, Teresa Raimondi^1*°^, Chiara De Gregorio^1^, Daria Valente^1,2^, Filippo Carugati^1^, Valeria Torti^1^, Olivier Friard^1^, Longondraza Miaretsoa^1,3^, Cristina Giacoma^1§^, Marco Gamba^1§^

affiliation and e-mail address of the corresponding author:

Anna Zanoli

anna.zanoli@unito.it

Department of Life Sciences and System Biology, University of Turin, Via Accademia Albertina 13, Torino, Italy

Teresa Raimondi

Teresa.raimondi@unito.it

Department of Life Sciences and System Biology, University of Turin, Via Accademia Albertina 13, Torino, Italy


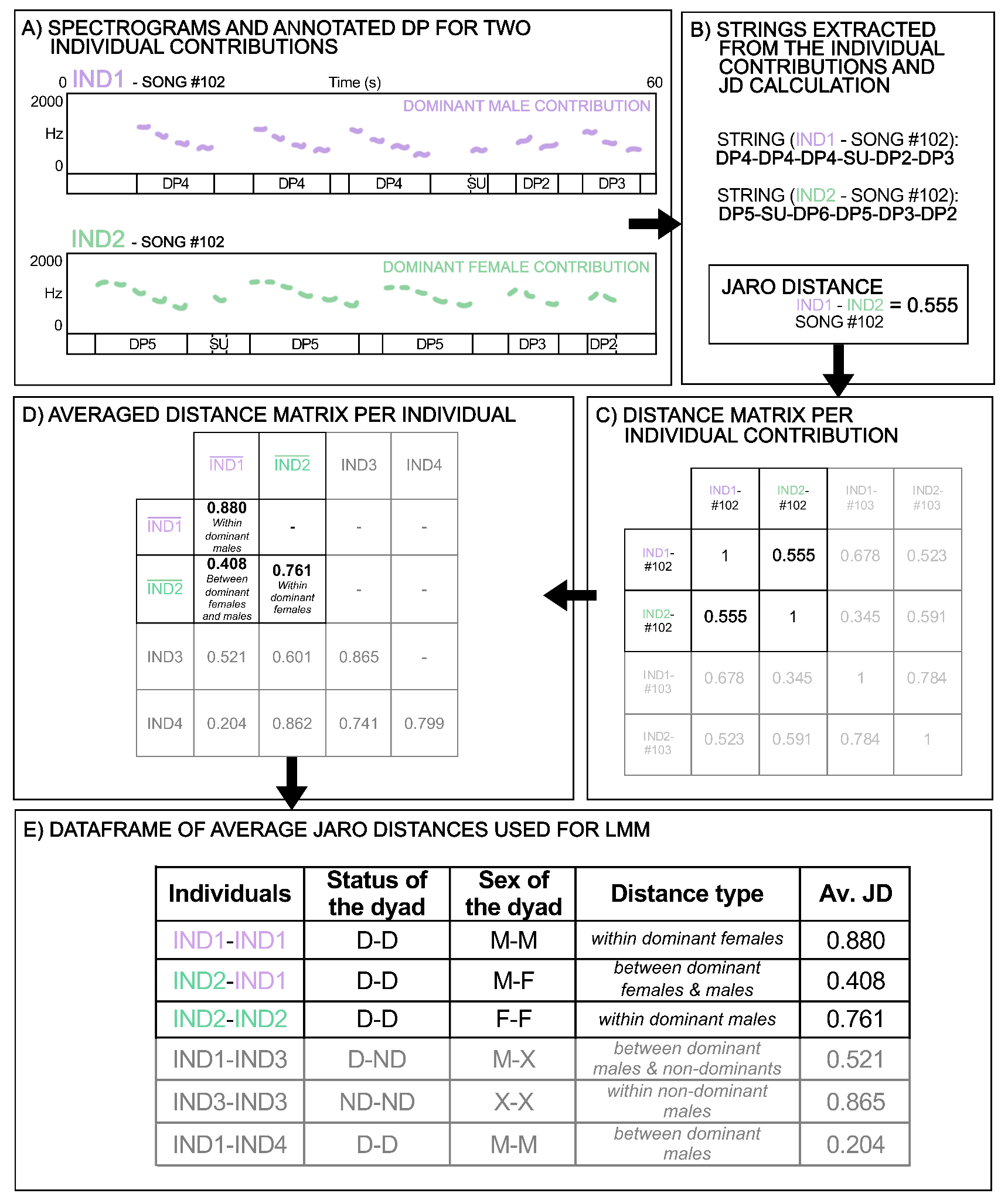


**Figure SM1 -** **A) Spectrograms and annotated Descending Phrases (DP) for two individual contributions**. The two spectrograms show the fundamental frequency of the modulated units, composing descending phrases, of two individuals: one dominant male (IND1 in purple) and one dominant female (IND2 in green) of a specific song (song code #102). Every DP is labeled with its phrase type, indicating the number of units composing it. **B) Strings extracted from the individual contributions and Jaro Distance (JD) calculation.** The string represents the concatenation of phrases extracted from every individual contribution, and the Jaro Distance is calculated between two strings (e.g. between the string of song #102 of IND1 and IND2). Below, the detailed calculation of the JD of the displayed exemple:

1. DP4 | DP4 | DP4 | SU | DP2 | DP3 s1=6 (the length of string 1)
2. DP5 | SU | DP6 | DP5 | DP3 | DP2 s2=6 (the length of string 2)

COMMON UNITS IN ORDER:

1. SU | DP2 | DP3
2. SU | DP3 | DP2

COUNT OF MATCHINGS (m) : 3 (SU, DP2, DP3)

COUNT OF NON-MATCHING AT THE SAME POSITION: 2 (DP2, DP3)

NUMBER OF TRANSPOSITION (t) : 1 (if in the first string we invert the position between DP2 and DP3 we obtain 2 identical strings)

$$JD= \frac{1}{3}\left( \frac{m}{s1}+ \frac{m}{s2}+\frac{m-t}{m} \right)$$

$$JD= \frac{1}{3}\left( \frac{3}{6}+ \frac{3}{6}+\frac{3-1}{3} \right)=0.555$$

**C) Distance matrix per individual contribution.** A distance matrix is extracted, showing all possible contrasts between all extracted strings in the sample. The specific example (IND1 and IND2 song #102) is highlighted in colors and bold black. **D) Averaged distance matrix per individual.** An averaged matrix is extracted, reporting the average JD per dyad of individuals (e.g. the average JD of all contrasts of all strings between IND1 and IND2). **E) Dataframe of average JD used for LMM.** From the averaged matrix, a data frame is extracted: it reports the individuals of the dyad, their status, sex, distance type and average JD. This dataframe is then used to build the LMM (with Average JD being the response variable).

| **contrast** | **estimate** | **SE** | **z-ratio** | **p-value** |
| --- | --- | --- | --- | --- |
| Between dominant females - Between dominant females & males (BDF - BDFDM) | -0.023 | 0.008 | -2.835 | 0.105 |
| **Between dominant females - Between dominant males (BDF - BDM)** | **0.039** | **0.009** | **4.150** | **0.001** |
| **Between dominant females - Between dominant females & non-dominants (BDF - BDFND)** | **-0.045** | **0.007** | **-6.207** | **<0.001** |
| Between dominant females - Between dominant males & non-dominants (BDF - BDMND) | -0.002 | 0.007 | -0.332 | 1.000 |
| **Between dominant females - Between non-dominants (BDF - BND)** | **-0.032** | **0.007** | **-4.457** | **<0.001** |
| Between dominant females - Within dominant females (BDF - WDF) | 0.025 | 0.015 | 1.641 | 0.782 |
| **Between dominant females & males - Between dominant males (BDFDM - BDM)** | **0.062** | **0.008** | **7.719** | **<0.001** |
| **Between dominant females & males - Between dominant females & non-dominants (BDFDM - BDFND)** | **-0.022** | **0.005** | **-4.225** | **0.001** |
| **Between dominant females & males - Between dominant males & non-dominants (BDFDM - BDMND)** | **0.020** | **0.005** | **3.815** | **0.004** |
| Between dominant females & males - Between non-dominants (BDFDM - BND) | -0.010 | 0.005 | -1.815 | 0.672 |
| **Between dominant males - Between dominant females & non-dominants (BDM - BDFND)** | **-0.084** | **0.007** | **-11.584** | **<0.001** |
| **Between dominant males - Between dominant males & non-dominants (BDM - BDMND)** | **-0.042** | **0.007** | **-5.709** | **<0.001** |
| **Between dominant males - Between non-dominants (BDM - BND)** | **-0.071** | **0.007** | **-9.862** | **<0.001** |
| Between dominant males - Within dominant males (BDM - WDM) | 0.042 | 0.015 | 2.837 | 0.105 |
| **Between dominant females & non-dominants - Between dominant males & non-dominants (BDFND- BDMND)** | **0.043** | **0.004** | **10.347** | **<0.001** |
| **Between dominant females & non-dominants - Between non-dominants (BDFND - BND)** | **0.013** | **0.004** | **3.176** | **0.040** |
| **Between dominant males & non-dominants - Between non-dominants (BDMND - BND)** | **-0.030** | **0.004** | **-7.343** | **<0.001** |
| **Between non-dominants - Within non-dominants (BND - WND)** | **0.048** | **0.010** | **4.957** | **<0.001** |

**Table SM1** Tukey-Kramer test between distances of dominant male and female and non-dominant indris, resulting from the LMM testing whether intrinsic individual features could affect JDs between individuals. P-values for multiple comparisons were corrected applying the Bonferroni correction (R-package “emmeans”; Russel 2021).

|  | **estimate** | **SE** | **df** | **t-value** | **p-value** |
| --- | --- | --- | --- | --- | --- |
| (Intercept) | -1.028 | 0.060 | a | -17.195 | a |
| Sex*Status | - | - | 2 | - | 0.009 |
| Sex*Status (Dominant male)^b,c^ | 0.156 | 0.084 | - | 1.863 | 0.072 |
| Sex*Status (Non-dominant)^b,c^ | 0.252 | 0.075 | - | 3.341 | 0.002 |

**Table SM2** Summary of the GLMM testing the effect of sex (for dominant individuals) and status (dominant vs. non-dominant) on individual contributions diversity. Full vs. null model: χ^2^=9.837, df=2, p-value=0.007. ^a^Not shown as not having a meaningful interpretation. ^b^Estimate ± SE refers to the difference in response between the reported level of this categorical predictor and the reference category of the same predictor. ^c^These predictors were dummy coded, with ‘Dominant female’ being the reference category.

| **COMPARISON** | **NORM DIVERSITY** | | **N** |
| --- | --- | --- | --- |
|  | ***Rho*** | ***p-value*** |  |
| 1MZ bevolo - jery | 0.491 | <0.001 | 108 |
| 2MZ soa - max | 0.450 | <0.001 | 102 |
| 3MZ mena - ratsy | 0.526 | 0.014 | 22 |
| 3MZa mena - mahagaga | 0.116 | 0.466 | 43 |
| 4MZ eva - koto | 0.326 | 0.028 | 46 |
| 5MZ fern - graham | 0.684 | 0.011 | 13 |
| 5MZa takona - graham | 0.425 | 0.002 | 52 |
| 6MZ befotsy - zokibe | 0.386 | 0.007 | 51 |
| 8MZ bemasoandro - jonah | 0.251 | 0.103 | 42 |
| 9MZ sissie - emilio | 0.291 | 0.069 | 40 |

**Table SM3** Results of the Spearman’s correlation tests performed between the normalized diversity values of each dominant female with the respective dominant male to test a possible covariation of duetting partners’ normalized diversity.

|  | **ID** | **GROUP** | **ENTROPY RATE** |
| --- | --- | --- | --- |
| **DOMINANT**  **MALES** | JERY | 1MZ | 1.666 |
|  | MAX | 2MZ | 1.592 |
|  | MAHAGAGA | 3MZa | 0.000 |
|  | RATSY | 3MZb | 1.972 |
|  | KOTO | 4MZ | 1.791 |
|  | GRAHAM | 5MZ | 1.484 |
|  | ZOKIBE | 6MZ | 1.417 |
|  | JONAH | 8MZ | 1.598 |
|  | EMILIO | 9MZ | 1.946 |
| **DOMINANT**  **FEMALES** | BEVOLO | 1MZ | 1.841 |
|  | SOA | 2MZ | 1.774 |
|  | MENA | 3MZ | 1.858 |
|  | EVA | 4MZ | 1.834 |
|  | FERN | 5MZa | 1.529 |
|  | TAKONA | 5MZb | 1.594 |
|  | BEFOTSY | 6MZ | 1.796 |
|  | BEMASOANDRO | 8MZ | 1.613 |
|  | SISSIE | 9MZ | 1.836 |
| **NON**  **-**  **DOMINANTS** | BERTHE | 1MZ | 0.949 |
|  | CAMY | 1MZ | 1.426 |
|  | FOTSY | 1MZ | 1.577 |
|  | AFO | 2MZ | 0.000 |
|  | FANIHY | 2MZ | 0.000 |
|  | TOVO | 2MZ | 1.582 |
|  | ANA | 3MZ | 1.116 |
|  | FALY | 3MZ | 1.731 |
|  | TONGA | 3MZ | 1.532 |
|  | GIBET | 4MZ | 1.788 |
|  | HENDRY | 4MZ | 1.847 |
|  | MEVA | 4MZ | 1.325 |
|  | VOARY | 5MZ | 1.886 |
|  | CESARE | 8MZ | 1.695 |
|  | EME | 8MZ | 1.320 |
|  | MIKA | 8MZ | 1.438 |
|  | ZAFY | 8MZ | 1.696 |
|  | BENY | 9MZ | 0.000 |
|  | DOSY | 9MZ | 1.584 |
|  | OVY | 9MZ | 1.766 |

**Table SM4** Entropy rate values for each individual, organized by dominant male, dominant female and non-dominants.
